# Phase II study of gefitinib in combination with docetaxel as first-line therapy in metastatic breast cancer

**DOI:** 10.1038/sj.bjc.6603141

**Published:** 2006-05-09

**Authors:** F Ciardiello, T Troiani, F Caputo, M De Laurentiis, G Tortora, G Palmieri, F De Vita, M R Diadema, M Orditura, G Colantuoni, C Gridelli, G Catalano, S De Placido, A R Bianco

**Affiliations:** 1 Cattedra di Oncologia Medica, Dipartimento Medico-Chirurgico di Internistica Clinica e Sperimentale ‘F Magrassi e A Lanzara’, Seconda Università degli Studi di Napoli, Via S Pansini 5, 80131 Naples, Italy; 2 Cattedra di Oncologia Medica, Dipartimento di Endocrinologia e Oncologia Molecolare e Clinica, Università degli Studi di Napoli ‘Federico II’, Naples, Italy; 3 Divisione di Oncologia Medica, Ospedale SG Moscati, Avellino, Italy

**Keywords:** metastatic breast cancer, taxanes, small-molecule EGFR tyrosine kinase inhibitors, combination therapy

## Abstract

We have evaluated the activity and safety of gefitinib, a small-molecule epidermal growth factor receptor (EGFR) tyrosine kinase inhibitor, in combination with docetaxel as first-line treatment of women with metastatic breast cancer (MBC). In total, 41 patients with MBC were enrolled in a first-line combination therapy study with oral gefitinib (250 mg day^−1^) and intravenous docetaxel (75 mg m^−2^, the first 14 patients; or 100 mg m^−2^, the following 27 patients, on day 1 of a 3-week cycle). Out of 41 patients, 38 received at least one cycle of therapy. There were no differences in activity or tolerability between the two docetaxel doses. G3/4 toxicities were neutropenia (49%), diarrhoea (10%), acne-like rash (5%), and anaemia (2%). Complete plus partial responses (CR+PR) were observed in 22 out of 41 patients with a 54% response rate (95% confidence interval (CI) 45–75%). The 22 patients that achieved a response following six cycles of docetaxel plus gefitinib continued gefitinib monotherapy (median duration, 24 weeks; range, 2–108+ weeks). Two patients with PR following combination therapy achieved a CR during gefitinib monotherapy. Complete plus partial responses correlated with oestrogen receptor (ER) status, since they occurred in 19 out of 27 (70%) patients with ER-positive tumours as compared to three out of 14 (21%) patients with ER-negative tumours (*P*=0.01).

Breast cancer is the most common malignancy among women and is the second leading cause of death for cancer in the Western countries after lung cancer. Although only a minority of patients is initially diagnosed with metastatic breast cancer (MBC), approximately 30–40% of all patients with early breast cancer, which are treated with curative intent will ultimately develop metastatic disease. Survival rates for MBC patients have been improved in recent years. However, despite advances in therapy, MBC largely remains an incurable disease and after documentation of metastasis the median survival time is approximately 2 years (Greenberg *et al*, 1996; Hortobagyi, 2002; Mincey and Perez, 2004). Docetaxel is a first-generation taxane that has a wide spectrum of antitumour activity and a number of unique characteristics compared to other chemotherapeutic agents, including the other taxane paclitaxel (Ringel and Horwitz, 1991; Riou *et al*, 1992; Bissery *et al*, 1995; Montero *et al*, 2005). Docetaxel is active in MBC patients (Nabholtz *et al*, 1999; Sjöström *et al*, 1999; Bonneterre *et al*, 2002; O’Shaughnessy *et al*, 2002; Tabernero *et al*, 2004; reviewed in Montero *et al*, 2005). In phase II studies docetaxel treatment determines response rates (RR) of approximately 38–68% (reviewed in Nabholtz and Gligorov, 2005). In phase III studies, when it is used as single agent in previously untreated women with MBC, docetaxel determines RR ranging between 30 and 48% (reviewed in Montero *et al*, 2005), whereas docetaxel in combination with an anthracycline determines a RR of 42–59% with a time to progression (TTP) ranging between 6.3 and 10.5 months (reviewed in Montero *et al*, 2005).

There is a large body of scientific evidence that the epidermal growth factor receptor (EGFR) pathway contributes to a number of processes involved in cancer cell proliferation, survival and invasion rendering it an attractive target for anticancer therapy (Ciardiello and Tortora, 2001). Studies in genetically modified mice have suggested a role for the EGFR pathway in mammary development and neoplastic transformation (Luetteke *et al*, 1999). In fact, overexpression of the EGFR ligand, transforming growth factor *α* (TGF*α*), in the mammary epithelium results in mammary hyperplasias and carcinomas after a prolonged latency (Matsui *et al*, 1990; Sandgren *et al*, 1990). Overexpression of EGFR is found in 14–91% of breast cancer and this has been correlated with disease progression and poor prognosis (Klijn *et al*, 1992; Fox *et al*, 1994; Klijn *et al*, 1994; Salomon *et al*, 1995).

There are several agents in clinical development that target the EGFR, and two of the most effective pharmacologic approaches currently under clinical investigation are small-molecule EGFR tyrosine kinase inhibitors and EGFR-blocking monoclonal antibodies (Ciardiello and Tortora, 2001; Grunwald and Hidalgo, 2003; Mendelsohn and Baselga, 2003). Gefitinib (ZD1839, Iressa™) is an orally active, small-molecule, reversible EGFR tyrosine kinase inhibitor (Herbst *et al*, 2004). Preclinical studies have shown that gefitinib has a broad spectrum of antitumour activity including human breast cancer (Ciardiello *et al*, 2000). Further, the combination of gefitinib with different cytotoxic drugs including docetaxel potentiates the antitumour activity of these drugs (Ciardiello *et al*, 2000; Sirotnak *et al*, 2000). In this respect, we have demonstrated that gefitinib is active and restores the sensitivity to docetaxel or to paclitaxel in multidrug-resistant, taxane-resistant human breast cancer cells (Ciardiello *et al*, 2002). Gefitinib is active also in breast cancer cell models which are resistant to endocrine therapy (Nicholson *et al*, 2002; Knowlden *et al*, 2003; Shou *et al*, 2004). In this respect, it has been shown that the EGFR-dependent autocrine pathway plays a key role both in intrinsic or de novo resistance to tamoxifen in ER positive, HER2 overexpressing MCF-7 breast cancer cells and in the acquired resistance to tamoxifen in tamoxifen-treated MCF-7 cells (Nicholson *et al*, 2002; Knowlden *et al*, 2003; Shou *et al*, 2004). In both experimental systems, gefitinib has a significant antitumour activity (Nicholson *et al*, 2002; Knowlden *et al*, 2003; Shou *et al*, 2004).

Based on these preclinical data, we have performed a phase II study of the combination of gefitinib and docetaxel as first-line treatment in patient with MBC. We have evaluated the safety, the tolerability profile and the clinical activity of gefitinib, 250 mg daily, in combination with docetaxel on a 3 weeks schedule at two different doses (75 mg m^−2^ and 100 mg m^−2^).

## PATIENTS AND METHODS

### Patients

Female patients aged 18 years or older with histologically confirmed MBC who had not previously received chemotherapy, hormonal therapy, immunotherapy or treatment with monoclonal antibodies for metastatic disease were eligible for this study. Patients were required to have measurable disease as defined in the Response Evaluation Criteria in Solid Tumours (RECIST) criteria and adequate general health status (Eastern Cooperative Oncology Group, ECOG, Performance Status, PS, 0–1). Patients who had received prior radiotherapy within the 2 weeks before entry into the trial were ineligible. Any patient with a history of a second malignancy within 5 years, with the exception of curatively treated basal cell carcinoma of the skin or cervical cancer *in situ*, was ineligible. Absence of severe and uncontrolled systemic disease such as unstable respiratory, cardiac, hepatic or renal disease was required. The following laboratory parameters documented within 1 week before enrollment were required: absolute neutrophil count (ANC) greater than 1.5 × 10^9^ l^−1^ (L) and platelets greater than 100 × 10^9^ l^−1^; ALT or AST⩽1.5 times the upper limit of normal range (ULRR) and alkaline phosphatase of ⩽2.5 times the ULRR; bilirurbin within normal limits and creatinine of ⩽1.5 times the ULRR. Women of childbearing potential should have had a negative pregnancy test before enrollment and were advised to practice appropriate contraception while on study. Patients were excluded from treatment with phenytoin, carbamazepine, barbiturates or rifampicin while on protocol. Concomitant bisphosphonates were allowed for patients with bone metastasis. This study was conducted in accordance with the Declaration of Helsinki and the study protocol was reviewed and approved by the local Research Ethics Committees (University of Naples and Ospedale SG Moscati, Avellino). Before study entry, each patient signed a written informed consent. Patients were enrolled between August 2002 and May 2004. Data analysis has been performed 12 months after the last patient had been enrolled (31 May 2005). The median follow-up was 23 months (range, 12–34 months).

### Treatment plan

Docetaxel and gefitinib combined treatment was planned for initial three cycles. Patients who had a clinical response (i.e. patients with complete response (CR), partial response (PR), or stable disease (SD)) after the first three clycles received additional three cycles of combination therapy. Gefitinib was continued as monotherapy only for patients who had CR or PR after the end of six cycle of combination therapy and this treatment was given until disease progression, unacceptable toxicity or withdrawal of consent. Patients received gefitinib, 250 mg, orally, once daily, continuously, in the morning at approximately the same time each day. The initial docetaxel dose was 75 mg m^−2^ as intravenous (i.v.) administration for 60 min, every 3 weeks. Docetaxel dose was escalated to 100 mg m^−2^ i.v. administration for 60 min, every 3 weeks, after the first 14 patients completed the planned treatment without the occurrence of any unacceptable toxicity as defined by an event that required dosage reduction or interruption in more than three patients. Patients received three doses of prophylactic dexamethasone, 8 mg i.m., every 12 h starting the 24 h before docetaxel infusion. Toxicities were graded according to the National Cancer Institute Common Toxicity Criteria (NCI-CTC), Version 2.0. Dose interruption was planned for NCI-CTC grade 3 and 4 toxicity related to gefitinib. Once the toxicity decreased in severity to grade 1, the patient may have continued on the assigned dose. Repeated dose interruptions were allowed as required, for a maximum of 14 days on each occasion. No dose reduction was allowed. If toxicity recurred after drug rechallenge, and further interruptions were considered insufficient to manage the toxicity, gefitinib discontinuation was required and patients were withdrawn from the trial. To manage docetaxel-related toxicity, treatment with docetaxel was delayed by no more than 2 weeks. If treatment was delayed for either haematologic or nonhaematologic toxicity for more than 2 weeks, the patients were withdrawn from the trial. In case of grade 2 neutropenia and of grade 3 thrombocytopenia, treatment with docetaxel was delayed until toxicity was resolved to grade 1 or less. In case of repeated delays due to grade 2 neutropenia, the dose of docetaxel could be reduced to 75 mg m^−2^ or to 50 mg m^−2^, for all the subsequent infusions in patients who received, respectively, the initial docetaxel dose of 100 mg m^−2^ or of 75 mg m^−2^. If further delays were required the patients would have been withdrawn from the trial. The treatment with docetaxel was planned to be discontinued with patients taken off study in case of grade 4 neutropenia for 7 days or more and/or grade 3–4 neutropenia with axilar fever ⩾38°C or documented infection.

### Patient clinical evaluation and assessment of response

Before study entry, a complete medical history, physical examination including performance status, height, weight and vital signs, electrocardiogram, urine analysis, pregnancy test if indicated, and radiographic tumour assessment within 21 days before start treatment were performed. Patients were evaluated at the start of each cycle during the combination treatment with physical examination, laboratory tests while adverse reactions were documented every week, whereas radiographic tumour assessment was repeated at the end of cycles 3 and 6. During gefitinib monotherapy, physical examination, haematology, biochemistry, evaluation of adverse events and radiographic tumour assessment were performed every 6 weeks until patient progression or trial closure.

### Study end points

The primary objective of this trial was to evaluate the activity of oral gefitinib administration in combination with docetaxel as first-line treatment in patients with MBC by estimating the objective tumour response rates (CR and PR) according to the RECIST criteria (Therasse *et al*, 2000). Secondary objectives were to estimate the disease control rate (CR, PR plus SD), the TTP, the overall survival (OAS) and to further characterise the safety and tolerability profile of gefitinib, 250 mg daily dose, alone and in combination with every 3 weeks i.v. docetaxel. Responses were evaluated by the investigators and were confirmed after 4 weeks.

### Statistical analysis

This was an open, noncomparative, two centre, phase II trial. The Fleming's method was used to calculate the number of patients required (Fleming, 1982). A sample size of 41 patients was calculated as sufficient to give an 80% probability of rejecting a baseline RR of 35% with an exact 5% one-sided significance test when the true response is at the clinically relevant rate of 55%. The hypothesis that the RR is equal or less than the baseline was considered to be rejected if 20 or more responses were observed in the 41 patients. All patients that were enrolled and received trial treatment were considered as the intention-to-treat (ITT) population. The analysis population for all efficacy end points were the ITT population. Response rates were estimated using the exact two-sided type 95% confidence interval (CI) method based on the binomial proportions. The association between the different clinicopathologic characteristics and responses to treatment was studied by the use of contingency tables. Statistical significance was evaluated by the Pearson *χ*^2^ test. Duration of response, TTP and OAS were estimated by the Kaplan–Meier method (Kaplan and Meier, 1958).

## RESULTS

### Patients characteristics

A total of 41 female patients with MBC, who had not previously received chemotherapy or hornomotherapy for metastatic disease, were enrolled in this study. The clinical characteristics of these women are listed in Table 1. The median age was 58 years; most women had a good ECOG PS, in particular 39 patients (95%) had a PS 0 at screening, while only two patients had a PS 1 (5%). The prevalent histotype was ductal infiltrating carcinoma (37 out of 41 patients, 90%), while four patients (10%) had a lobular infiltrating carcinoma. The majority of women (83%) had visceral disease. Five of the patients on study (12%) had HER-2 3+ at the immunohistochemistry screening of the primary tumour, 22 (54%) patients had a HER-2-negative tumour, whereas the HER-2 status of 14 (34%) patients was not known. Tumours in 27 patients were oestrogen receptor-positive (66%) and in 14 cases (34%) were ER negative. Most patients had received, in the adjuvant setting, prior chemotherapy and hormonotherapy (tamoxifen, 20 mg daily planned for 5 years) (73 and 59%, respectively). The 24 patients that had received adjuvant hormonotherapy therapy were treated with tamoxifen for 6 months to 5 years (median, 3 years). Of the patients, 22 (54%) had been treated with antracyclines and six patients (15%) had received a taxane in the adjuvant setting.

### Toxicity

A total of 216 cycles (median, 5.2. cycles; range, 1–6) of docetaxel infusions were administrated. None of the patients had docetaxel dose delay and no woman developed toxicity requiring dose adjustment of docetaxel. The first 14 patients were treated for up to six cycles with the combination of docetaxel (75 mg m^−2^ dose) and gefitinib. Since no major toxicity occurred, as per protocol planning, the following 27 patients enrolled in this study were treated with combination of docetaxel (100 mg m^−2^ dose) and gefitinib. There were no differences in grade and profile of toxicity between the two docetaxel doses. Therefore, toxicity data are presented on the whole 41 patient population. Thirty eight patients received at least one cycle of combination therapy with docetaxel and gefitinib. In general, treatment was well tolerated. The worst grades of treatment-related toxicity for the 41 patients are listed in Table 2. Three patients developed, despite the required premedication with dexamethasone, hypersensitivity reactions after a few minutes of starting the first i.v. infusion of docetaxel. These women, who were never treated with gefitinib, were taken off protocol and switched to a chemotherapy regimen without docetaxel. Haematologic toxicity was noted in the majority of patients during the combination treatment period with grade 3 or 4 neutropenia affecting 49% of patients. Neutropenia was the only grade 4 haematologic toxicity observed. No patients developed neutropenic fever. Grade 3 anaemia occurred in one patient. There was minimal thrombocytopenia. Nonhaematologic toxicities related to study treatment are also listed in Table 2. Gastrointestinal side effects such as nausea/vomiting or stomatitis were mild with only grade 1 or 2 toxicity recorded. Grade 3 diarrhoea occurred in four patients (10%) and was controlled with loperamide administration. Only one patient with grade 3 diarrhoea refused further treatment after one cycle of combination therapy. Neuropathy affected 17% of patients and none exceeded grade 2 toxicity. Several patients developed asthenia, but only two with grade 3 toxicity. Abnormal values for ALT and/or AST were reported in 11 patients, but only in two patients reached grade 3 toxicity. Two patients had grade 3 acne-like rash after the first cycle of combination therapy. After 1 week of gefitinib treatment suspension and recover to grade 1 skin toxicity, treatment with gefitinib was restarted. In these two patients, the intensity of this skin reaction did not exceed grade 2 with the following cycles of combination treatment. However, two of the three patients who experienced grade 2 skin toxicity decided to withdraw their consent after one cycle of combination therapy and were switched to a different chemotherapy regimen.

### Clinical activity

Of 41 patients enrolled in this study, three were never treated with gefitinib, because they had to stop the first adminstration of i.v. docetaxel on day 1 of the first cycle without receiving gefitinib due to hypersensitivity reactions to docetaxel. On an intention to treat analysis of the entire 41 patient population, major clinical responses were observed in 22 patients for an overall RR of 54% (CI, 45–75%) (Table 3). Five CR and 17 PR were observed. Two of the 17 patients with PR following completing the six cycles of docetaxel–gefitinib combination therapy achieved a CR during gefitinib monotherapy. Six patients (14%) had a SD and 13 patients (32%) experienced disease progression. The 22 patients that achieved a response following six cycles of docetaxel plus gefitinib continued gefitinib monotherapy (median duration, 24 weeks; range, 2–108+ weeks). Major responses (CR+PR) were evaluated in correlation with all clinical and pathologic characteristics (listed in Table 1) of the patients. No significant correlation was observed between the achievement of a clinical response and any parameter which was evaluated, except for a statistically significant association between response to treatment and ER status (Table 4). In fact, in those patients with ER-positive tumours, CR+RP were observed in 19 out of 27 (70%) patients, whereas among patients with ER-negative cancers the RR was three out of 14 (21%) patients. If we consider only the 35 patients who were treated with gefitinib and docetaxel combination for at least two cycles of therapy, major clinical responses (CR+RP) were observed in 19 out of 23 (82%) patients with ER-positive tumours, whereas among patients with ER-negative cancers the RR was three out of 12 (25%). The median OAS in the entire population of 41 patients enrolled in this study could not be calculated at the time of the analysis for the low numbers of deaths occurred. The median TTP which was analysed on an intention to treat basis on the all 41 patients was 8.2 months. The median duration of response for responding patients was 6.5 months (range, 3–23.5+ months).

## DISCUSSION

To our knowledge this is the first report on docetaxel and gefitinib combination as first-line treatment of patients with MBC. The results of this study suggest that treatment with docetaxel and gefitinib is an active and generally well-tolerated regimen in women with MBC who have not been previously treated for metastatic disease. Most of the toxicities observed were consistent with those expected when using docetaxel as single agent (reviewed in Montero *et al*, 2005). The most frequent G3/4 toxicity was neutropenia, although no febrile neutropenia or neutropenic infection occurred. Peripheral neuropathy and asthenia observed in this trial were also similar to those observed with single agent docetaxel given every 3 weeks (reviewed in Montero *et al*, 2005). The adverse events associated with gefitinib were diarrhoea and acne-like rash and were in line with the reported safety profile for this drug (Herbst *et al*, 2004). In particular, two patients experienced grade 3 skin toxicity. However, after 1 week of treatment suspension, gefitinib was restarted and the intensity of this skin reaction did not exceed grade 2 thereafter.

The RR reported in this trial are in a range which is comparable to those obtained when docetaxel is combined with antracyclines (reviewed in Montero *et al*, 2005). Several studies have been published on the combination of docetaxel with trastuzumab, the first therapeutic agent targeting the human epidermal growth factor receptor-2 (HER2) which has been used in breast cancer (Baselga *et al*, 1996). Trastuzumab is currently used in association with several chemotherapy drugs, including taxanes, for women who have been selected for having HER2 overexpressing MBC. In particular, several phase II studies of trastuzumab in combination with docetaxel, as first- or as second-line treatment for patients with HER2 – overexpressing MBC, have been reported with RR ranging from 45 to 72% (Burris, 2001; Esteva *et al*, 2002; Montemurro *et al*, 2004; Tedesco *et al*, 2004). More recently, the results of a randomised phase II trial evaluating the activity and the efficacy of trastuzumab plus docetaxel in the first-line treatment of HER2 – overexpressing MBC patients have been reported (Marty *et al*, 2005). In this study, a 61% RR, a 11.7 months TTP and a 31.2 months median OAS have been observed.

A potentially interesting observation which emerges from our study is that a statistically significant association has been found between clinical response to gefitinib plus docetaxel treatment and ER status (Table 4). In fact, in those patients with ER-positive tumours, the RR is 70%, whereas among patients with ER-negative cancers the RR is 21%. The majority of patients who experienced SD or PD had ER-negative tumours. These clinical data may be explained by the recent experimental findings in MCF-7 human breast cancer cells, which are initially oestrogen-dependent for growth and sensitive to the antioestrogen tamoxifen. MCF-7 cells which are chronically cultured in the presence of tamoxifen generally become tamoxifen-resistant between 3 and 6 months (Nicholson *et al*, 2002; Knowlden *et al*, 2003). Tamoxifen-resistant MCF-7 cells exhibit markedly increased levels of EGFR and HER-2 as compared to tamoxifen-sensitive parental MCF-7 cells (Nicholson *et al*, 2002; Knowlden *et al*, 2003). Furthermore, these cells exhibit an increased dependence on EGFR signaling for proliferation and survival and they become extremely sensitive to gefitinib (Nicholson *et al*, 2002). Therefore, a possible interpretation for the findings of our study is that women with ER-positive-tumours, which have been treated chronically up to 5 years in the adjuvant setting with the antioestrogen tamoxifen, may have eventually developed a MBC in which the EGFR-driven pathway has become important for cancer cell growth and for such reason become sensitive to gefitinib. In this context, the EGFR signaling pathway which is upregulated in tamoxifen-resistant ER-positive breast cancer would be of critical relevance in the escape from the growth inhibition induced by the antioestrogen tamoxifen. The possibility that women with ER-positive breast cancer may be selected for the treatment with EGFR inhibitors is supported also by the preliminary results of a phase II study in which it has been investigated the activity and safety of gefitinib in patients with tamoxifen-resistant ER-positive breast cancer (Robertson *et al*, 2003). In this trial, 66% of patients with tamoxifen-refractory disease achieved a clinical benefit (PR plus SD) with gefitinib monotherapy, as compared to only 11% of patients with ER-negative disease. On the contrary, three phase II studies in unselected, heavily pretreated women with MBC have shown that gefitinib monotherapy has minimal clinical activity with RR ranging between 0 and 2% (Albain *et al*, 2002; Baselga *et al*, 2005; von Minckwitz *et al*, 2005). However, a randomised phase II trial has recently shown that a short 4- to 6-weeks treatment with gefitinib monotherapy or with gefitinib plus the aromatase inhibitor anastrozole in postmenopausal breast cancer patients with ER-positive and EGFR-positive tumours before curative surgery determines PR in 12 out of 22 patients and in 14 out of 28 patients, respectively (Polychronis *et al*, 2005). These findings suggest a direct antiproliferative effect of gefitinib in this untreated breast cancer patient population.

In summary, the results of this study suggest that treatment with gefitinib in combination with docetaxel is a potentially active and well-tolerated regimen in untreated patient with MBC. A clinically relevant issue is the identification of potential predictive factors, which could help to select breast cancer patients who could respond to anti-EGFR targeted therapies. The results of this study suggest that women with ER-positive tumours have a higher RR and are most likely the patients that could benefit from the docetaxel plus gefitinib combination. However, further clinical translational research studies are necessary to define the role of gefitinib alone and/or in combination with docetaxel in the management of this subset of MBC patients.

## Figures and Tables

**Table 1:** Patient characteristics

	**Patients (*N*=41)**	
	***N***	**%**
*Age (years)*		
Median	58	
Range	40–75	
		
*ECOG performance status*		
0	39	95
1	2	5
2	0	0
		
*Histology*		
Ductal infiltrating carcinoma	37	90
Lobular infiltrating carcinoma	4	10
		
*ER expression (IHC)*		
Positive	27	66
Negative	14	34
		
		
*HER-2 expression (IHC)*		
3+	5	12
Negative	22	54
Unknown	14	34
		
*Prior adjuvant therapy*		
Yes	30	73
No	11	27
		
Prior adjuvant chemotherapy	30	73
Prior adjuvant anthracyclines	22	54
Prior adjuvant taxanes	6	15
Prior adjuvant hormonotherapy (tamoxifen)	24	59
		
*Dominant sites of disease*		
Visceral	34	83
Soft tissue (lymph nodes and bone sites)^a^	7	17

a No patient had only bone metastases.

**Table 2:** Worst grade of toxicity for the 41 patients

	**Grade**							
	**1**		**2**		**3**		**4**	
**Toxicity**	**Patients**	**%**	**Patients**	**%**	**Patients**	**%**	**Patients**	**%**
Anaemia	7	17	4	10	1	2	0	
Thrombocytopenia	2	5	0		0		0	
Neutropenia	12	29	4	10	6	15	14	34
Nausea	8	20	4	10	0		0	
Vomiting	5	12	2	5	0		0	
Stomatitis	2	5	2	5	0		0	
Diarrhoea	9	22	5	12	4	10	0	
Mucositis	7	17	2	5	0		0	
Pain	1	2	0		0		0	
Fever	2	5	5	12	0		0	
Hypersensitivity^a^	0		0		3	7	0	
Neuropathy	6	15	1	2	0		0	
Acne-like rash	4	10	3	7	2	5	0	
Asthenia	6	15	4	10	2	5	0	
Increase in transaminases	6	15	3	7	2	5	0	

a Hypersensitivity reactions after few minutes of starting the first intravenous injection of docetaxel were observed in three patients, which, therefore, were never treated with gefitinib and which were switched to a chemotherapy regimen without docetaxel. Toxicities were recorded according to the National Cancer Institute Common Toxicity Criteria (NCI-CTC) version 2.0.

**Table 3:** Response to therapy for the 41 patients

	**Patients (*N*=41)**	
**Response**	***N***	**%**
Overall response	22	54
Complete response (CR)	5	12
Partial response (PR)	17	42
		
Stable disease (SD)	6	14
		
Progressive disease (PD)	13	32

Among these 41 patients, three were never treated with gefitinib, because they had to stop the first adminstration of i.v. docetaxel on day 1 of the first cycle before receiving gefitinib due to hypersensitivity reactions to docetaxel. Three additional patients refused further therapy after one cycle. Refusal was due to grade 3 diarrhoea in one patient and to grade 2 acne-like rash in the other two patients. For the intention to treat analysis, all six patients were recorded as patients with progressive disease.

**Table 4:** Correlation between ER positivity and major clinical responses (CR+PR) to therapy

**Responses**	**ER positive (27/41 patients)**	**ER negative (14/41 patients)**
CR+PR (22/41 patients)	19/27 patients (70%)	3/14 patients (21%)
SD+PD (19/41 patients)	8/27 patients (30%)	11/14 patients (79%)

Pearson's *χ*^2^ test: *P*=0.01.
